# Tissue transglutaminase exacerbates renal fibrosis via alternative activation of monocyte-derived macrophages

**DOI:** 10.1038/s41419-023-05622-5

**Published:** 2023-03-02

**Authors:** Yoshiki Shinoda, Hideki Tatsukawa, Atsushi Yonaga, Ryosuke Wakita, Taishu Takeuchi, Tokuji Tsuji, Miyako Tanaka, Takayoshi Suganami, Kiyotaka Hitomi

**Affiliations:** 1 Cellular Biochemistry Lab., Graduate School of Pharmaceutical Sciences, Nagoya University, Tokai National Higher Education and Research System, Furo-cho, Chikusa, Nagoya, 464-8601 Japan; 2 Department of Molecular Medicine and Metabolism, Research Institute of Environmental Medicine, Nagoya University, Tokai National Higher Education and Research System, Furo-cho, Chikusa, Nagoya, 464-8601 Japan; 3 Department of Immunometabolism, Nagoya University Graduate School of Medicine, Tokai National Higher Education and Research System, Furo-cho, Chikusa, Nagoya, 464-8601 Japan; 4 Institute of Nano-Life-Systems, Institutes of Innovation for Future Society, Nagoya University, Tokai National Higher Education and Research System, Furo-cho, Chikusa, Nagoya, 464-8601 Japan

**Keywords:** Cell polarity, Post-translational modifications, Chronic inflammation, Transcriptomics, Chronic kidney disease

## Abstract

Macrophages are important components in modulating homeostatic and inflammatory responses and are generally categorized into two broad but distinct subsets: classical activated (M1) and alternatively activated (M2) depending on the microenvironment. Fibrosis is a chronic inflammatory disease exacerbated by M2 macrophages, although the detailed mechanism by which M2 macrophage polarization is regulated remains unclear. These polarization mechanisms have little in common between mice and humans, making it difficult to adapt research results obtained in mice to human diseases. Tissue transglutaminase (TG2) is a known marker common to mouse and human M2 macrophages and is a multifunctional enzyme responsible for crosslinking reactions. Here we sought to identify the role of TG2 in macrophage polarization and fibrosis. In IL-4-treated macrophages derived from mouse bone marrow and human monocyte cells, the expression of TG2 was increased with enhancement of M2 macrophage markers, whereas knockout or inhibitor treatment of TG2 markedly suppressed M2 macrophage polarization. In the renal fibrosis model, accumulation of M2 macrophages in fibrotic kidney was significantly reduced in TG2 knockout or inhibitor-administrated mice, along with the resolution of fibrosis. Bone marrow transplantation using TG2-knockout mice revealed that TG2 is involved in M2 polarization of infiltrating macrophages derived from circulating monocytes and exacerbates renal fibrosis. Furthermore, the suppression of renal fibrosis in TG2-knockout mice was abolished by transplantation of wild-type bone marrow or by renal subcapsular injection of IL4-treated macrophages derived from bone marrow of wild-type, but not TG2 knockout. Transcriptome analysis of downstream targets involved in M2 macrophages polarization revealed that ALOX15 expression was enhanced by TG2 activation and promoted M2 macrophage polarization. Furthermore, the increase in the abundance of ALOX15-expressing macrophages in fibrotic kidney was dramatically suppressed in TG2-knockout mice. These findings demonstrated that TG2 activity exacerbates renal fibrosis by polarization of M2 macrophages from monocytes via ALOX15.

## Introduction

Macrophages can play beneficial or detrimental roles in several diseases, depending on their activation status in the pathological tissue microenvironment [1]. Macrophages can polarize into at least two major subtypes, classically activated (M1) and alternatively activated (M2), each of which plays an important role in the opposing regulation of inflammatory progression and suppression in chronic inflammation diseases [2–4]. The surrounding environment that governs macrophage function is closely related to the specific function of macrophages: M1 is activated by lipopolysaccharide/interferon γ and exhibits proinflammatory features, whereas M2 is activated by IL-4/IL-13 stimulation and displays anti-inflammatory properties. However, if injury is uncontrolled and M2 macrophage activity persists, these cells can be detrimental to tissue homeostasis. Excessive activation of M2 macrophages regulates the continuous production of growth factors such as TGF-β, which promotes myofibroblast proliferation and activation, resulting in extracellular matrix deposition as seen in tissue fibrosis diseases [2–4].

Chronic kidney disease (CKD) affects more than 840 million people globally and is characterized by structural abnormalities and dysfunction of the kidneys that last for more than three months [5, 6]. This persistent renal damage causes excessive activation of myofibroblasts and multiple immune cells, especially macrophages, leading to tubulointerstitial fibrosis. Renal fibrosis is a common pathway for pathological deterioration from CKD to end-stage renal failure, but because the detailed pathogenesis mechanism has not been elucidated, there are currently few effective therapeutic agents. M2 macrophage activation correlates with the progression of renal fibrosis. Deletion of macrophages in a unilateral ureteral obstruction (UUO)-treated mouse tubulointerstitial fibrosis model suppressed renal fibrosis, suggesting that M2 macrophages are involved in the development of fibrosis [7, 8]. Indeed, M2 macrophages secrete high levels of TGF-β and promote epithelial-to-mesenchymal transition and subsequent tubulointerstitial fibrosis [9–12]. Furthermore, the existence of fibrosis-specific monocyte/macrophage has been reported [13], increasing the importance of analyzing the pathogenesis of fibrosis with a focus on macrophages. However, studies on M2 macrophages in mice cannot still not be directly applied to humans because mouse M2 macrophages have different basic characteristics, such as cell surface antigen markers.

Martinez et al. previously identified tissue transglutaminase (TG2) as the only reproducible M2 macrophage marker common to both humans and mice by both transcriptomic and proteomic analyses [14]. TG2 is a Ca^2+^-dependent protein crosslinking enzyme that catalyzes the formation of covalent bond between the γ-carboxamide groups of glutamine residues in peptide bonds and various primary amines, including the ε-amino group of lysine residues in target proteins [15–17]. TG2 expression and crosslinking activity may be associated with differentiation of monocytes and functional maturation of macrophages [18–24]. Additionally, TG2 is involved in cell adhesion and migration of monocytes [23, 25]. However, the detailed role of TG2 in the induction of M2 macrophage and the relevance to fibrosis remains unclear.

In renal fibrosis, TG2 is involved in the accumulation of fibrous proteins through crosslinking and stabilization of extracellular matrix proteins such as collagen and fibronectin, and pathogenesis of renal fibrosis is suppressed in TG2-knockout (TG2KO) and TG2 inhibitor-treated mice [26–28]. This study supports our hypothesis that TG2 promotes renal fibrosis via induction of M2 macrophage polarization infiltrated into kidney. Therefore, the current study sought to elucidate the mechanism by which TG2 regulates the polarization of M2 macrophages and the subsequent function of TG2-induced M2 macrophages in the pathogenesis of renal fibrosis and to better understand the pathogenesis of renal fibrosis, which still lacks useful therapeutic strategies.

Here, we reveal that macrophage polarization is a major contributor to the mechanism whereby TG2 induces fibrosis and that TG2 has a crucial role for the polarization of M2 macrophages of mouse and human origin. TG2-dependent M2 macrophage polarization was found to be derived from bone marrow cells and caused renal fibrosis. Furthermore, we found that TG2 is required for induction of an arachidonate lipoxygenase, leading to polarization of M2 macrophages. These studies may help develop new therapeutic targets not only for renal fibrosis, but also for diseases involving macrophage such as atherosclerosis and osteoporosis, as well as various inflammatory, neurodegenerative, and autoimmune diseases.

## Materials and methods

### Materials

Chemical reagents were mainly purchased from WAKO chemicals (Osaka, Japan) and Nacalai Tesque (Kyoto, Japan). Primary and fluorescein-conjugated secondary antibodies were listed in Suppl. Table S1. Polyclonal anti-TG2 antibody was produced in our laboratory [29]. HRP-conjugated secondary antibodies were obtained from Jackson ImmunoResearch Laboratories (West Grove, PA, USA). Cystamine was obtained from Sigma-Aldrich (St. Louis, USA). Z-DON and Boc-DON were obtained from Zedira (Darmstadt, Germany). PD146176 and 15S-hydroxy-5Z,8Z,11Z,13E-eicosatetraenoic acid, 15(S)-HETE, were purchased from Cayman Chemical (Ann Arbor, MI, USA).

### Ethics statement

Animal experiments were conducted at Nagoya University, complying with the national guidelines for the care and use of laboratory animal. All animal experiments were approved by the animal care and use committee of Nagoya University (No. P220002). All animal experiments were performed under anesthesia and all efforts were made to minimize suffering.

### Animal experiments

C57BL/6J male mice (8–12 weeks) were purchased from Japan SLC Inc (Shizuoka, Japan) and group-housed with food and water available *ad libitum*. TG2 knockout and enhanced GFP-transgenic mice were kindly provided by Dr. Robert M. Graham (Victor Chang Cardiac Research Institute, Australia) [30] and Dr. Masaru Okabe (Osaka University, Osaka, Japan) [31], respectively.

### Unilateral ureteral obstruction

The unilateral ureteral obstruction (UUO) was performed according to the method described by Shweke et al. [26]. Briefly, under the anesthesia with 2% isoflurane, the left ureter was ligated at two separated points. Sham-operated mice had their ureter exposed but not ligated. Mice after UUO surgery were perfused with PBS to remove the blood in kidney, and pieces of the kidney were either fixed in 4% paraformaldehyde for histological examination. Cystamine was orally administrated at 1.86 mg/kg/day two days before UUO surgery.

### Histological analysis

Cryosections from the kidney (5 μm) were fixed with 4% paraformaldehyde and reacted with anti-TG2, F4/80, α-SMA, and ALOX15 antibodies. The specific signal was detected by the fluorescent-dye-conjugated secondary antibody. As a negative control, the primary antibody was replaced with the same amount of non-immune IgG (NI-IgG) from rabbit or rat (Sigma-Aldrich). Collagen fibers were detected using picrosirius red (Wako chemicals). Briefly, kidney sections (10 μm) were fixed in a saturated solution of picric acid with formalin and acetic acid for 15 min and then stained with 0.05% sirius red reagent. In the sections from each animal, more than 5 randomly selected microscopic fields were captured by a Keyence BZ-9000 microscope. All images were quantitatively estimated for collagen fibers in picrosirius red staining within the respective kidney area according to the tutorial about “quantifying stained tissue” in image analyzer (Image J software, National Institute of Health, Bethesda, MD, USA). Each red color image was split as grayscale images and thresholded optimally. The positive areas above threshold level were measured and an average of at least 3 field from four replicates in each sample group was determined.

### Flow cytometric analysis

Kidneys were cut and digested in Hanks’ buffered saline solution (HBSS) containing 1 mg/ml collagenase (Wako) and 50 µg/ml DNase I (Roche). After filtering through a 70 µm mesh, cells were washed, incubated with the antibodies listed in Suppl. Table S1, and analyzed using Attune Acoustic Focusing Cytometer and Attune Cytometric Software v2.1.0.8626 (Life technologies).

### Quantitative real-time PCR

Total RNA was extracted from cultured cells using the Sepasol-RNA Super Reagent (Nacalai Tesque). Corresponding cDNA were prepared using ReverTra Ace qPCR RT Master Mix with gDNA Remover kit (TOYOBO, Osaka, Japan) and Real-time PCR analysis was performed using THUNDERBIRD SYBR qPCR Mix (TOYOBO) in a LightCycler 96 (Roche Diagnostics, Mannheim, Germany). Used specific primer pairs were summarized in Suppl. Table S2.

### Western blotting analysis

The cell lysates were homogenized in lysis buffer containing 50 mM Tris-HCl (pH 8.0), 150 mM NaCl, 5 mM EDTA, 1% NP-40, 1 mM NaF, phosphatase inhibitor, and protease inhibitor cocktail (Merck Millipore). After centrifugation, supernatants were collected, and their protein concentrations were measured by Bradford assay (Bio-rad). Then, these samples were mixed with SDS-containing buffer, boiled, subjected to sodium dodecyl sulfate-polyacrylamide gel electrophoresis (SDS-PAGE), and transferred to polyvinylidene difluoride membrane (Merck Millipore). After blocking with PBS containing 5% skim milk or BSA, the membrane was reacted with primary antibody listed in Suppl. Table S1, and the specific signal was detected by the peroxidase-conjugated secondary antibody and chemiluminescence reagent (Thermo Scientific, IL, USA). Each experiment was conducted in triplicate.

### Bone marrow transplantation experiments

Bone marrow transplantation experiments were performed as reported [32]. In brief, bone marrow cells obtained from donor mice were washed three times with cold PBS and injected intravenously (3 × 10^6^ cells) into 8.5 Gy-irradiated 8-week-old male recipient mice. After 4 weeks, the substitution rate of bone marrow cells was determined by counting EGFP-positive cells in the peripheral blood, and then the mice were subjected to UUO experiments. WT and TG2KO mice were also transplanted with each bone marrow from WT and TG2KO mice, and then subjected to UUO experiments.

### Macrophage cell culture

Bone marrow-derived macrophages (BMDMs) were prepared according to the method reported previously [33]. Briefly, bone marrow cells were isolated from femur and tibia of 6–10 weeks male mice and differentiated for 6 days in RPMI medium containing 10% FBS and conditioned medium from L929 fibroblasts. M2 macrophage polarization was induced by 20 ng/ml murine recombinant IL-4 (PeproTech, Rocky Hill, NJ, USA) in 5% FBS containing RPMI medium after serum starvation. Human monocytic leukemia cell line (THP-1) was differentiated into macrophages by incubation with 150 nM phorbol 12-myristate-13-acetate (PMA; AdipoGen Life Sciences) for 24 h. M2 macrophage polarization was induced by 20 ng/ml recombinant human IL-4 (PeproTech) in 5% FBS containing RPMI after starvation. For TG2 knockdown, targeting siRNA (sense 5′-cccugaucguugggcugaatt-3′ and antisense 5′-uucagcccaacgaucagggtt-3′) and MISSON siRNA universal negative control #1 (SIC-001) purchased from Sigma-Aldrich were used as reported previously [34].

### RNA sequence analysis

Total RNA from THP-1 cells was lysed and extracted using tissue total RNA mini kit according to manufacturer’s instructions. After the QC procedures, total RNAs were deposited for transcriptome analysis (Filgen biosciences and nanoscience, Nagoya Japan). Briefly, mRNA was enriched using oligo(dT) beads and rRNA was removed. First, the mRNA was fragmented randomly by adding fragmentation buffer, then the cDNA was synthesized by using mRNA template and random hexamers primer, after which a custom second-strand synthesis buffer (Illumina), dNTPs, RNase H, and DNA polymerase I were added to initiate the second-strand synthesis. Second, after a series of terminal repair, a ligation, and sequencing adaptor ligation, the double-stranded cDNA library was completed through size selection and PCR enrichment. Sequences were performed on NovaSeq6000 (Illumina), (6GB/PE150). Raw reads were aligned to the human genome (hg38) using the RNA-Seq Alignment App on Basespace (Illumina, CA). The data reported in this paper have been deposited in the Gene Expression Omnibus database (accession no. GSE222284).

Perseus software (version 1.6.14.0) was used to determine the genes differentially and significantly identified in four sample groups treated with vehicle, IL-4, Z-DON, and IL-4 plus Z-DON. A total of 38,552 genes with more than 50 of read counts detected between groups (*n* = 3) were analyzed. Differential gene expressions (DGEs) were determined using the threshold (FDR < 0.01). The gene list of both 2-fold filtered significant DGEs between vehicle- vs IL-4-treated samples and 1.5-fold filtered significant DGEs between IL-4 vs IL-4 plus Z-DON-treated samples were selected (90 genes). Among them, 55 and 35 genes with TG2-dependent increase and decrease were identified, respectively. The heat maps and hierarchical clustering were generated using Morpheus (https://clue.io/morpheus).

### Statistical analyses

Quantitative data are expressed as the means plus the standard deviation of three replicates from at least three independent experiments. The statistical significance of differences was assessed using Student’s *t*-test and the values of *P* < 0.05 were considered to indicate statistical significance. The one-way ANOVA with post hoc Tukey’s multiple comparisons test was performed with EZR (Saitama Medical Center, Jichi Medical University, Saitama, Japan), which is a graphical user interface for R (The R Foundation for Statistical Computing, Vienna, Austria) [35]. No randomization blinding was used in animal experiments.

## Results

### Macrophage infiltration was reduced in the renal fibrosis model of TG2KO mice

**Fig. 1:**
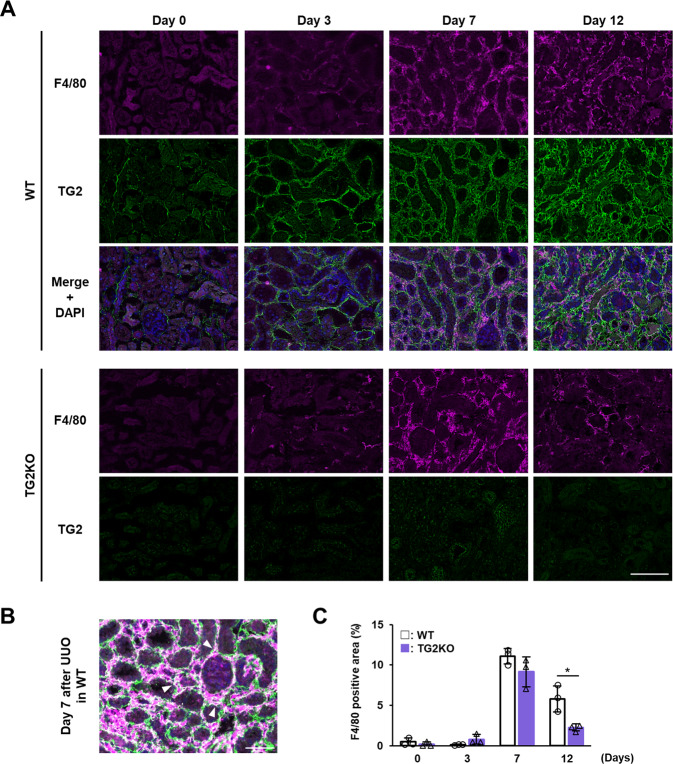
Evaluation of macrophage infiltration into the kidney after UUO surgery in TG2KO mice. Mice were conducted to UUO surgery. Kidney sections from WT and TG2KO mice after UUO surgery were fixed in 4% paraformaldehyde and immunostained using anti-F4/80 plus Alexa Fluor 594 anti-rat antibodies and anti-TG2 plus Alexa Fluor 488 anti-rabbit antibodies (**A**). The nuclei were counterstained with DAPI. Scale bar = 100 μm. The merged image from WT on day 7 after UUO was enlarged (**B**). Arrowheads indicate the similar distributions between F4/80 and TG2. Scale bar = 50 μm. The percentages of F4/80-positive area are presented (**C**). Data are presented as the mean ± SD (*n* = 3) (*P < 0.05, Student’s *t* test).

We initially evaluated the correlation between the TG2 expression level and macrophage infiltration into the kidney in a renal fibrosis model using UUO surgery. Fluorescent immunostaining revealed that TG2 expression was markedly enhanced in the interstitial area 3 days after UUO and remained strongly increased until day 12 (Fig. 1A). The expression of macrophage marker F4/80 was highest on day 7 and partly colocalized with TG2, suggesting that TG2 is expressed in some macrophages during renal fibrosis progression (Fig. 1B). TG2KO mice had fewer F4/80-positive areas compared to WT mice at 12 days after UUO, and a similar tendency was observed at 7 days after UUO, but not significantly different (Fig. 1C). These results suggest that TG2 plays an important role in macrophage infiltration and accumulation at the relatively late stages of renal fibrosis (>day 7) and may account for the increase in M2 macrophages in the late response rather than affecting M1 macrophages in the early response.

### TG2 is required for M2 macrophages infiltrating fibrotic kidney

**Fig. 2:**
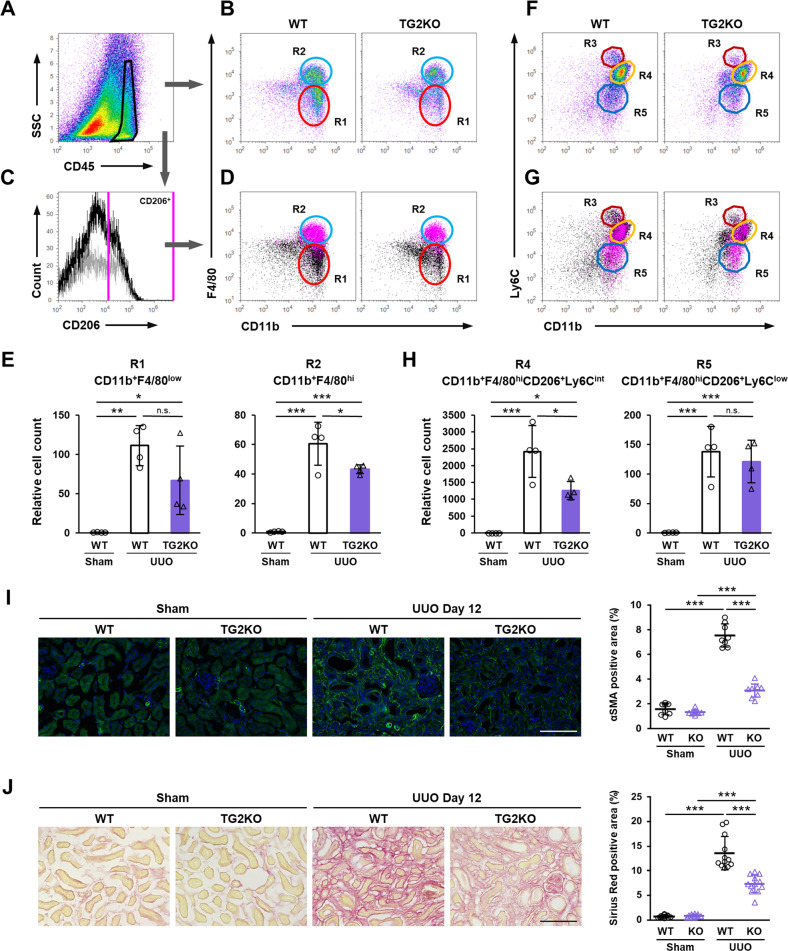
Characterization of infiltrated macrophage into fibrotic kidney after UUO surgery. CD45-positive cells from fibrotic kidney in WT and TG2KO mice were divided (**A**) into the CD11b^+^ F4/80^low^ (R1) and CD11b^+^ F4/80^hi^ (R2) groups (**B**). SSC side scatter. CD206-positive cells from WT (black) and TG2KO (gray) mice were selected in histogram plot (**C**) and colored with magenta in the dot plots shown in **B** (**D**). The relative cell counts of two groups were indicated (**E**). CD45-positive cells were also divided into the CD11b^+^ Ly6C^hi^ (R3), CD11b^+^ Ly6C^int^ (R4), and CD11b^+^ Ly6C^low^ (R5) groups (**F**). CD206-positive cells were colored with magenta in the dot plots shown in **F** (**G**). The relative cell counts of R4 and R5 groups gated by both F4/80^hi^ and CD206^+^ were indicated (**H**). Myofibroblasts in kidney sections were stained using anti-α-SMA antibody plus AlexaFluor 488 goat anti-rabbit IgG and the percentages of their positive area are presented (**I**). The nuclei were counterstained with DAPI. The collagen fibers in kidney sections were detected by picrosirius red staining and the percentages of their positive area are presented (**J**). Scale bars = 100 μm. Representative results in at least three independent samples were shown. (*P < 0.05, **P < 0.01, ***P < 0.001 by one-way ANOVA with post hoc Tukey’s multiple comparisons test).

We next used flow cytometry to investigate the infiltrated macrophage subtypes that decreased in abundance in the kidney of TG2KO mice after UUO. CD45-positive cells were gated from whole kidney cells (Fig. 2A) and then evaluated for CD11b and F4/80 expressions to yield two main population groups (Fig. 2B). CD45-positive CD11b^+^ F4/80^low^ (R1) and CD11b^+^ F4/80^hi^ (R2) cells were classically defined as M1 and M2 macrophages infiltrating the UUO-treated kidney, respectively (Fig. 2B) [36]. This is consistent with flow cytometry analysis of CD206^+^ cells that were backgated to the magenta-colored R2 group in Fig. 2C, D. The number of cells in the R2 group was significantly decreased in TG2KO mice comparison to WT mice, although that cell number in the R1 group was no significant different (Fig. 2B, E). Furthermore, when CD45^+^ cells were assessed for CD11b and Ly6C expressions, three population groups, CD45^+^ CD11b^+^ Ly6C^hi^ (R3), Ly6C^int^ (R4), and Ly6C^low^ (R5), were observed (Fig. 2F). CD206-positive M2 macrophages were classified in both the R4 and R5 groups but not in the R3 group (Fig. 2G). The comparison of each macrophage subtype revealed that the abundance of cells in the R4 group gated by both F4/80^hi^ and CD206^+^ was significantly decreased in TG2KO mice (Fig. 2H), suggesting that TG2 was involved in the polarization of the CD45^+^ CD11b^+^ F4/80^hi^ CD206^+^ Ly6C^int^ M2 macrophage subtype during renal fibrosis. Consistent with these results, the number of α-SMA-positive myofibroblasts and the level of collagen deposition detected by picrosirius red staining were also significantly suppressed in TG2KO mice (Fig. 2I, J).

### TG2 expression in bone marrow cells contributes to renal fibrosis after UUO

**Fig. 3:**
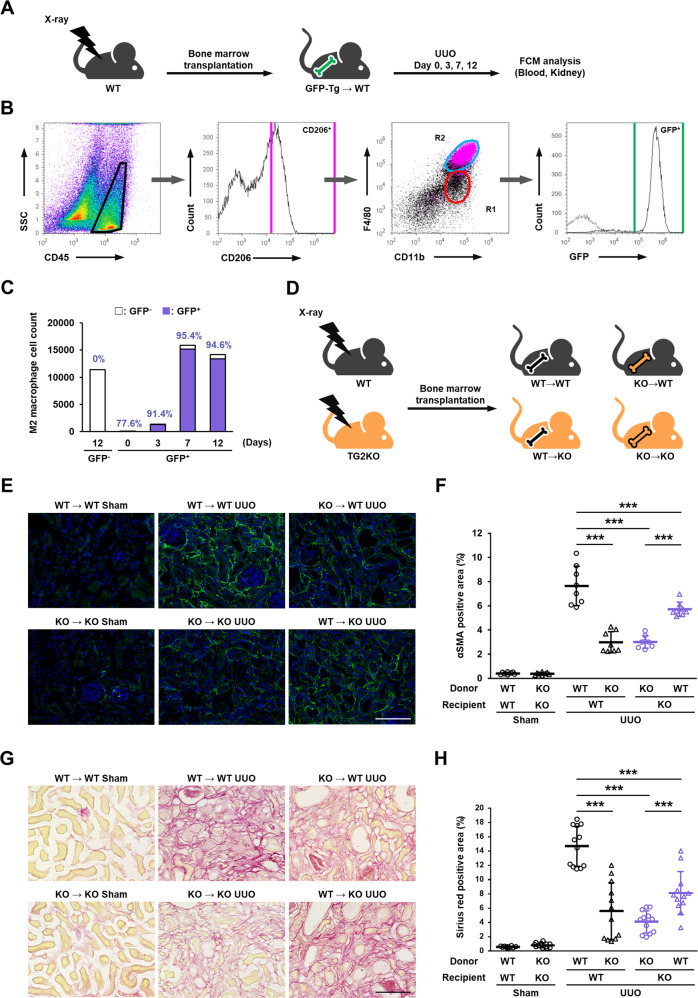
Role of TG2 in bone marrow-derived cells in renal fibrosis after UUO surgery. Mice were irradiated at lethal dose (8.5 Gy) of X-rays and transplanted with bone marrow cells isolated from GFP-transgenic mice by tail vein injection. After 4 weeks recovery period, mice were subjected to UUO surgery and analyzed on indicated days (**A**). The CD45-positive cells in fibrotic kidney were divided into F4/80 and CD11b, and these population classified by CD206 were colored with magenta (**B**). Then, the counts and percentages of GFP-positive cells in R2 group were analyzed and plotted (**C**). WT and TG2KO mice were lethally irradiated by X-rays and transplanted with bone marrow cells isolated from WT and TG2KO mice by tail vein injection (**D**). After 4 weeks recovery period, mice were conducted to UUO and analyzed on 14 days after UUO surgery. The myofibroblasts and collagen fibers in kidney sections were detected by immunofluorescence staining using anti-α-SMA antibody (**E**) and picrosirius red staining (**G**), respectively, and the percentages of their positive area are presented (**F**, **H**). The nuclei were counterstained with DAPI. Scale bars = 100 μm. Representative results in at least three independent samples were shown. (***P < 0.001 by one-way ANOVA with post hoc Tukey’s multiple comparisons test).

Monocyte-derived and resident macrophages have been reported to be involved in CKD [37, 38], and therefore we next performed bone marrow transplantation experiments to distinguish immune cell origin and confirm the correlation with TG2 expression. Mice were exposed to a lethal dosage of X-rays and then transplanted with bone marrow cells from GFP-transgenic mice via tail vein injection. After 4 weeks of recovery, UUO was performed on the mice, and cell counts analyzed at each indicated time after surgery (Fig. 3A). The percentage of GFP-positive cells in peripheral blood cells indicated the successful replacement in this transplantation model. This showed that >98% of CD45^+^ cells in blood cells were GFP^+^-cells derived from donor GFP-transgenic mice (Suppl. Fig. S1). We next examined whether the increased M2 macrophages in fibrotic kidney expressed GFP. The M2 macrophage population (CD45^+^ CD11b^+^ F4/80^hi^; R2) was confirmed by CD206 expression and almost exclusively expressed GFP (Fig. 3B). The abundance of these GFP-positive R2 populations markedly increased on days 7 and 12 after UUO compared with that on day 3 and were mostly derived from bone marrow cells (Fig. 3C). We next evaluated the levels of renal fibrosis using four experimental groups of bone marrow-chimeric mice transplanted in WT and TG2KO mice as indicated in Fig. 3D. WT and TG2KO mice were exposed to X-rays and transplanted with bone marrow cells from WT or TG2KO mice. In WT-recipient mice, mice harboring TG2KO bone marrow cells had significantly decreased numbers of myofibroblasts and the levels of renal fibrosis compared with levels in mice harboring WT bone marrow cells (Fig. 3E, G, upper column; Fig. 3F, H, lanes 3 vs. 4). Conversely, in TG2KO-recipient mice, mice harboring WT bone marrow cells had significantly increased numbers of myofibroblasts and the levels of renal fibrosis compared with levels in mice harboring TG2KO bone marrow cells (Fig. 3E, G, lower column; Fig. 3F, H, Lanes 5 vs. 6). The mice transplanted with TG2KO bone marrow cells had markedly suppressed fibrosis regardless of whether the recipient was WT or TG2KO (Fig. 3F, H, lanes 4 and 5), indicating that TG2-dependent induction of bone marrow-derived CD11b^+^ F4/80^hi^ CD206^+^ M2 macrophage subtypes may promote the pathogenesis of renal fibrosis.

### TG2 activity contributes to IL-4-induced M2 macrophage polarization and exacerbates renal fibrosis

**Fig. 4:**
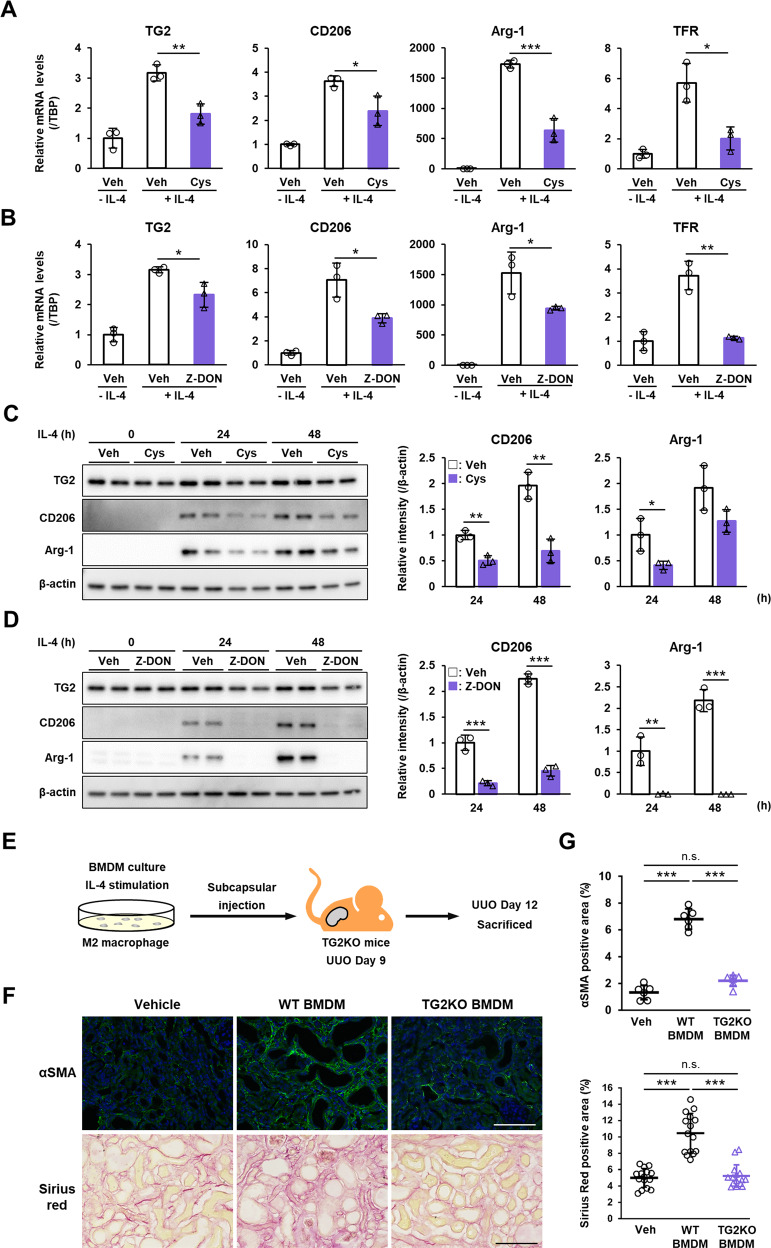
Role of TG2 in M2 macrophage polarization induced by IL-4 and renal fibrosis. BMDMs were prepared using bone marrow cells isolated from mice and cultured with L929 fibroblast conditioned medium. M2 macrophage polarization was induced by treatment of 20 ng/ml recombinant mouse IL-4 in the presence or absence of 0.4 mM cystamine and 50 μM Z-DON for 24–48 h. mRNA expression levels of TG2 and indicated mouse M2 macrophage markers were analyzed (**A**, **B**). Data were normalized against mRNA expression of TATA-binding protein (TBP) and relative values (a ratio of the control sample) were presented as the mean ± SD (*n* = 3) (**P < 0.01, *P < 0.05, Student’s *t* test). Veh Vehicle, Cys cystamine. The protein levels of these samples were analyzed by immunoblotting using the indicated antibodies (**C**, **D**). Anti-β-actin antibody was used as a loading control for each sample. Total intensities of all the bands in each sample were presented after normalizing the results to the expression levels in β-actin. The full-length blots with molecular mass markers are presented in Suppl. Figs. S2 and S3. BMDMs prepared from WT or TG2KO mice were treated by IL-4 for 2 h and transferred into renal subcapsule (4.75 × 10^5^ cells/mouse) of TG2KO mice on day 9 after UUO. These mice were sacrificed on day 12 after UUO and analyzed (**E**). The myofibroblasts and collagen fibers in kidney sections were detected by immunofluorescence staining using anti-α-SMA antibody and picrosirius red staining, respectively (**F**), and the percentages of their positive area are presented (**G**). The nuclei were counterstained with DAPI. Scale bars = 100 μm. Representative results in at least three independent samples were shown (***P < 0.001 by one-way ANOVA with post hoc Tukey’s multiple comparisons test).

Based on the above results, we speculated that TG2 expression in macrophages may contribute to polarization of M2 macrophages. In in vitro studies using bone marrow-derived macrophages (BMDMs), IL-4 treatment markedly increased in mRNA expression of TG2 and mouse M2 macrophage markers such as CD206, arginase (Arg)-1, transferrin receptor (TFR; Fig. 4A, B), whereas treatment with TG2 inhibitor such as cystamine and Z-DON significantly suppressed the induction of these M2 markers (Fig. 4A, B). Similar results were obtained in the protein levels of TG2 and M2 macrophage markers (Fig. 4C, D and Suppl. Figs. S2 and S3). Then, we next examined whether TG2-dependent M2 polarization in BMDMs was involved in the renal fibrosis. IL-4-treated BMDMs from WT or TG2KO mice were injected into renal subcapsule of TG2KO mice after UUO (Fig. 4E). Interestingly, the number of myofibroblasts and the levels of renal fibrosis in TG2KO mice were significantly increased by injection of BMDMs from WT mice, although there was no effect on renal fibrosis following injection of BMDMs from TG2KO mice (Fig. 4F, G). This was similar to the more severe renal fibrosis effect produced with TG2KO-recipient mice harboring WT bone marrow cells than that in mice harboring TG2KO bone marrow cells (Fig. 3E, G, lower column; Fig. 3F, H, Lane 5 vs. 6).

### Cystamine suppressed the M2 macrophage infiltrating fibrotic kidney

**Fig. 5:**
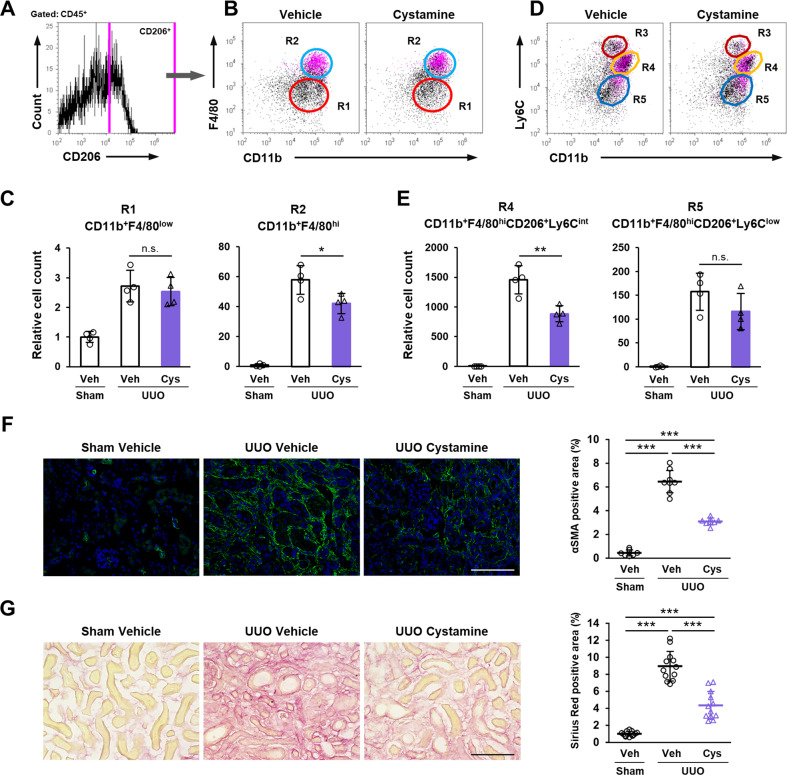
Effect of cystamine administration in M2 macrophage polarization and renal fibrosis. Mice were conducted to UUO surgery and orally administrated with cystamine (1.86 mg/kg/day). Renal CD45-positive cells were classified by CD206 (**A**) and colored with magenta in the dot plots divided into the CD11b^+^ F4/80^low^ (R1) and CD11b^+^ F4/80^hi^ (R2) groups (**B**). The relative cell counts of R1 and R2 groups were indicated (**C**). These cells also divided into CD11b^+^ Ly6C^hi^ (R3), CD11b^+^ Ly6C^int^ (R4), and CD11b^+^ Ly6C^low^ (R5) groups (**D**). The relative cell counts of R4 and R5 groups gated by F4/80^hi^ and CD206^+^ were indicated (**E**). The myofibroblasts and collagen fibers in kidney sections were detected by immunofluorescence staining using anti-α-SMA antibody (**F**) and picrosirius red staining (**G**), respectively, and the percentages of their positive area are presented in the graph on the right. The nuclei were counterstained with DAPI. Scale bars = 100 μm. Representative results in at least three independent samples were shown. *P < 0.05, **P < 0.01, ***P < 0.001 by one-way ANOVA with post hoc Tukey’s multiple comparisons test.

We next investigated whether the inhibition of TG2 activity could suppressed M2 macrophage polarization in an in vivo study. Oral administration of cystamine significantly decreased the abundance of M2 macrophages (CD45^+^ CD11b^+^ F4/80^hi^; R2) but not that of M1 macrophages (CD45^+^ CD11b^+^ F4/80^low^; R1; Fig. 5A–C). Cystamine administration also significantly decreased the abundance of CD11b^+^ CD206^+^ Ly6C^int^ M2 macrophages (R4), but not that of CD11b^+^ CD206^+^ Ly6C^low^ M2 macrophages (R5) (Fig. 5D). The comparison of each macrophage subtype revealed that the abundance of the R4 group gated by both F4/80^hi^ and CD206^+^ was significantly decreased in cystamine-treated mice after UUO (Fig. 2E), suggesting that cystamine inhibits the polarization of CD45^+^ CD11b^+^ F4/80^hi^ CD206^+^ Ly6C^int^ M2 macrophage subtype during renal fibrosis. Consistently, the number of myofibroblasts and the levels of renal fibrosis were also significantly suppressed by cystamine-treated mice (Fig. 5F, G).

### TG2 promotes the polarization of human M2 macrophages via intracellular crosslinking activity

**Fig. 6:**
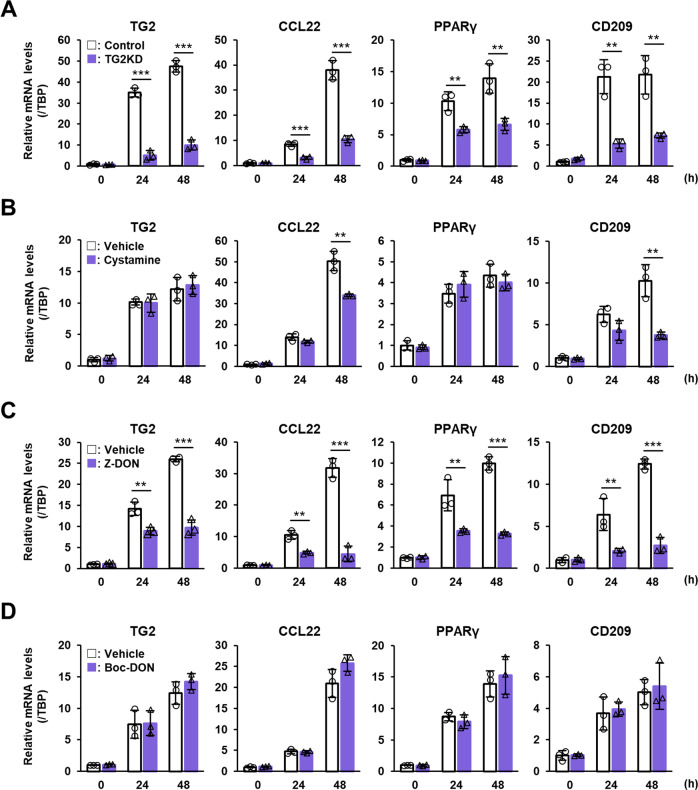
Role of TG2 in human M2 macrophage polarization induced by IL-4. Human monocytic leukemia cell line, THP-1, was treated with 150 nM PMA for 24 h. M2 macrophage polarization was induced by 20 ng/ml recombinant human IL-4. mRNA expression levels of TG2 and indicated human M2 macrophage markers were analyzed. For knockdown experiment, PMA-treated macrophages were transfected with siRNA against TG2 (**A**). As a negative control, scrambled siRNA (Control) was replaced with the same amount of TG2 siRNA. For the TG2 inactivation, cell-permeable (0.4 mM Cystamine, **B**; 50 μM Z-DON, **C**) and impermeable inhibitors (100 μM Boc-DON; **D**) were used. Data were normalized against mRNA expression of TBP and relative values were presented as the mean ± SD (*n* = 3) (***P < 0.001, **P < 0.01, *P < 0.05, Student’s *t* test).

TG2 was identified as the only marker common to mouse and human M2 macrophages based on both transcriptomics and proteomics [14], and we therefore, next examined the contribution of TG2 expression and activity in human M2 macrophages. The human monocyte leukemia cell line, THP-1, was differentiated into macrophages using PMA and then polarized into M2 macrophages by IL-4 treatment. Similar to the results using mouse BMDMs, IL-4 treatment markedly increased in mRNA expression of TG2 and human M2 macrophage markers such as C-C motif chemokine ligand 22 (CCL22), peroxisome proliferator-activated receptor γ (PPARγ), and CD209 (Fig. 6). The induction of these M2 markers were significantly inhibited by treatment with TG2 siRNAs (Fig. 6A and Suppl. Fig. S4) and cell-permeable inhibitors such as cystamine (Fig. 6B) and Z-DON (Fig. 6C), but not by the cell-impermeable inhibitor Boc-DON (Fig. 6D). These results suggest that the crosslinking activity of intracellular TG2 in macrophages plays a common role in promoting polarization of both human and mouse M2 macrophages.

### TG2 regulates M2 macrophage polarization via ALOX15 induction

**Fig. 7:**
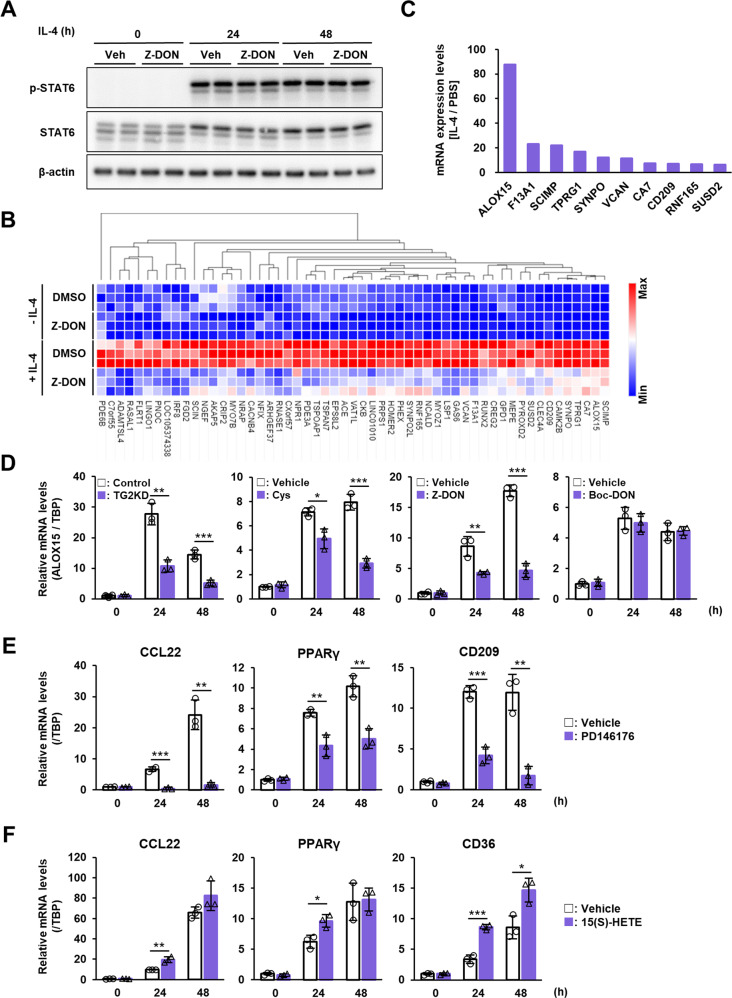
Analysis of molecular mechanism of M2 macrophage polarization that is regulated by TG2. Human macrophages differentiated from THP-1 were treated with 20 ng/ml IL-4 in the presence or absence of 50 μM Z-DON. Cells were extracted and analyzed by immunoblotting using the indicated antibodies (**A**). The full-length blots are presented in Suppl. Fig. S5. Total RNAs were conducted to transcriptome analysis as described in “Materials and methods” section. DGEs between vehicle- vs IL-4-treated samples (2-fold, FDR < 0.01) and between IL-4- vs IL-4 plus Z-DON samples (1.5-fold, FDR < 0.01) were determined. Among them, 55 genes that increase in TG2-dependent manner were identified and indicated as heat map (**B**). The relative means of the three groups of mRNAs whose expression was enhanced by IL-4 treatment among the 55 genes are plotted in ascending order (**C**). mRNA expression levels of ALOX15 were evaluated in the IL-4-treated human macrophages transfected with control and TG2 siRNA and treated with cystamine, Z-DON, and Boc-DON (**D**). mRNA expression levels of TG2 and indicated M2 macrophage markers were analyzed in IL-4-treated human macrophage combined treatment with 5 μM PD146176 (**E**) or 24 μM 15(S)-HETE (**F**). Data were normalized against mRNA expression of TBP and relative value were presented as the mean ± SD (*n* = 3) (***P < 0.001, **P < 0.01, *P < 0.05, Student’s *t* test).

To elucidate the reason why the TG2 knockdown and inactivation suppressed M2 macrophage polarization, we next investigated the molecular mechanism underlying this. We first checked the signal transducer and activator of transcription 6 (STAT6), upstream regulator in the signaling pathway induced by IL-4. IL4-treated human macrophages had significantly increased levels of phosphorylated STAT6 but were not affected by combined treatment with Z-DON (Fig. 7A and Suppl. Fig. S5), suggesting that STAT6 is phosphorylated upstream of TG2. We next performed global expression analysis via RNA sequencing to explore genes whose expression was regulated by TG2 activity. We selected 55 genes whose expression was altered by IL-4 treatment in a TG2-dependent manner (i.e., both significantly increased by more than 2-fold in IL-4 treatment and decreased by more than one-third by treatment in combination with Z-DON) (Fig. 7B). Expression of the arachidonate 15-lipoxygenase (*ALOX15*) gene was remarkably increased by IL-4 treatment (Fig. 7C) and was reduced by 40% by inhibition of TG2 activity (*data not shown*). As ALOX15 has been reported as a regulator of M2 macrophage polarization [39, 40], we next confirmed whether the ALOX15 expression was regulated by TG2 expression and activity and promoted TG2-dependent M2 macrophage polarization. The mRNA expression levels of ALOX15 were decreased in the IL-4-treated macrophages in combination with TG2 siRNA, cystamine, or Z-DON, but not with Boc-DON treatment (Fig. 7D and Suppl. Fig. S4). In addition, treatment with an inhibitor of ALOX15 activity, PD146176, decreased the mRNA expression of human M2 macrophage markers (Fig. 7E). Furthermore, treatment with the ALOX15 metabolite, 15(S)-HETE, significantly increased mRNA expression of M2 macrophage markers including CD36 in IL-4-treated macrophages (Fig. 7F). These results suggested that TG2 promoted M2 macrophage polarization via the expression and activity of ALOX15.

### ALOX15 expression was enhanced in macrophages infiltrating fibrotic kidney

**Fig. 8:**
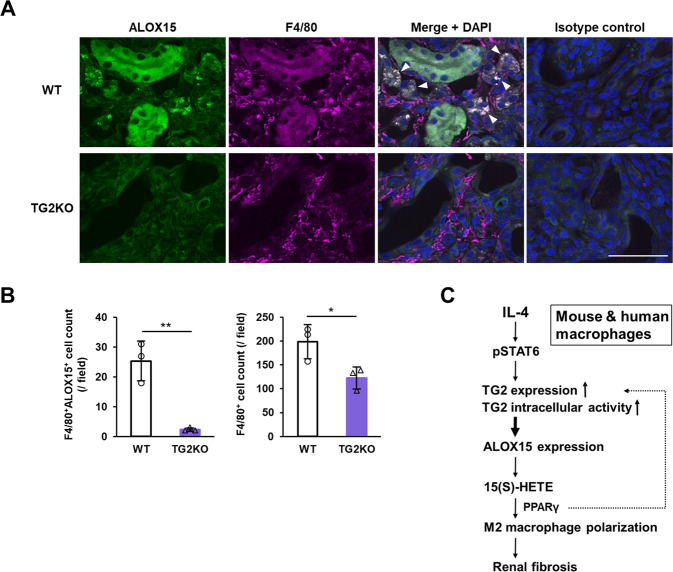
Evaluation of ALOX15 distribution in fibrotic kidney and its association with renal fibrosis. Kidney section on day 12 after UUO surgery were fixed in 4% paraformaldehyde and immunostained using anti-F4/80 plus Alexa Fluor 594 anti-rat antibodies and anti-ALOX15 plus Alexa Fluor 488 anti-rabbit antibodies (**A**). As an isotype control, each primary antibody was replaced with the same amount of rat and rabbit NI-IgG. The nuclei were counterstained with DAPI. Arrowheads indicate the similar distributions between ALOX15 and F4/80. Scale bar = 50 μm. Representative results in at least three independent samples were shown. The numbers of F4/80^+^ ALOX15^+^ cells and F4/80^+^ cells infiltrating kidney of WT and TG2KO mice after UUO were presented (**B**). Data are presented as the mean ± SD (*n* = 3) (**P < 0.01, *P < 0.05, Student’s *t* test). Schematic showing the molecular mechanism by which TG2 causes M2 polarization via ALOX15 expression and its metabolism in mouse and human macrophages, leading to renal fibrosis (**C**).

We finally evaluated whether ALOX15 expression was observed in macrophages infiltrating fibrotic kidney after UUO. Fluorescent immunostaining revealed that ALOX15 expression was enhanced in the F4/80^+^ macrophages in fibrotic kidney (Fig. 8A). Unexpectedly, expression of ALOX15 occurred not only in macrophages but also in renal tubules and glomeruli. Surprisingly, the number of ALOX15-positive F4/80^+^ macrophages was drastically reduced by approximately 90% in TG2KO mice (Fig. 8B, left panel) although the overall number of infiltrating F4/80^+^ macrophages was only reduced by approximately 40% in TG2KO mice as in Fig. 1C (Fig. 8B, right panel). Given that mice deficient in ALOX15 or treated with an ALOX15 inhibitor significantly suppressed the renal fibrosis by UUO [41], these results suggest that ALOX15 expression is enhanced by intracellular TG2 activity and induces M2 macrophage polarization, leading to pathological exacerbation of renal fibrosis.

## Discussion

In this study, we found that TG2 promotes both human and mouse M2 macrophage polarization via its intracellular crosslinking activity. In addition, the monocyte-derived TG2-induced M2 macrophages contribute to the pathogenesis of mouse renal fibrosis. Furthermore, we found that, at least in part by mechanism, TG2 markedly exacerbates renal fibrosis though the enhanced expression of ALOX15 and a metabolite derived from ALOX15 activity. First, we found that in TG2KO mice, there was only a significant decrease in the number of macrophages in the late stage of fibrotic kidney after UUO surgery (Fig. 1). This was interesting as TG2 had been previously thought to be mainly involved in the stabilization and accumulation of fibrous protein through crosslinking activity in renal fibrosis [42, 43], suggesting a new molecular mechanism whereby TG2 is involved in renal fibrosis.

The increased abundance of macrophages in fibrotic kidney was mainly derived from bone marrow but not from proliferative renal resident macrophages (Fig. 3C). The majority of macrophages are distributed throughout the body before birth and are capable of self-renewal and there is consequently little need for monocytes in adults under normal circumstances [44]. However, during a rapid response to inflammation, circulating monocytes must adhere to and infiltrate vascular endothelial cells adjacent to the injured tissue and then differentiate into macrophages [45]. TG2 is present in monocytes [20] and has been reported to contribute to adhesion and migration of monocytes on fibronectin [23]. Our results clearly indicated that TG2 expression in monocytes derived from bone marrow cells increased the abundance of M2 macrophage in fibrotic kidney, exacerbating renal fibrosis (Figs. 3D–H and 4E–G). Furthermore, both F4/80 and ALOX15 positive cells in fibrotic kidney were reduced by 90% in TG2KO mice although F4/80 positive cells were reduced by about half (Fig. 8). These suggested that loss of TG2 was associated with polarization of M2 macrophages rather than macrophage infiltration (Fig. 8B). However, it cannot be completely ruled out that TG2 is involved in the monocyte infiltration into the fibrotic kidney. This is because fibrosis was more severe in the renal subcapsular injection of BMDMs from WT mice into TG2KO mice than in the transplantation of bone marrow from WT mice into TG2KO mice, whereas the renal subcapsular injection itself may have contributed to worsening fibrosis. Similar to the results obtained in TG2KO mice, inhibitors for TG2 crosslinking activity predominantly reduced the amount of M2 macrophage in fibrotic kidney and suppressed renal fibrosis, despite the limitation of in vivo experiments that cystamine is not a specific inhibitor of TG2 activity alone (Fig. 5). In addition, in vitro studies using both BMDMs and PMA-treated THP-1 demonstrated that TG2 is a critical regulator for human and mouse M2 macrophage polarization (Figs. 4 and 6, and Suppl. Fig. S4). Although TG2 extracellularly contributes to activation of TGF-β [26, 46–48], which is a major regulator for cell differentiation, it was interesting to note that only intracellular, but not extracellular, TG2 regulated M2 macrophage polarization. Active inhibitors targeting TG2 are expected to contribute to the development of useful drugs for the regulation of pathogenesis associated with M2 macrophages [49].

Transcriptome analysis further revealed that ALOX15 expression was strongly induced by IL-4 in a TG2 activity dependent manner. Although ALOX15 was not identified as a gene/protein commonly induced in both mouse and human M2 macrophages [14], but has been reported as a highly inducible IL-4/IL-13 target gene in both mouse [40] and human [39]. ALOX15 belongs to a family of dioxygenases that convert unsaturated fatty acids, preferably arachidonic acid, into monoxide derivatives such as 15(S)-HETE [50, 51]. ALOX15 is implicated in many pathological processes and was recently reported to induce inflammation and fibrogenesis in mice after UUO [41]. Macrophage infiltration and renal fibrosis were demonstrated to decrease in both ALOX15-knockout and inhibitor-treated mice but were increased in transgenic ALOX15-overexpressing mice [41], and although that study did not include an in vitro analysis and the results from human macrophages, these results are consistent with our results obtained here. Additionally, studies of mice undergoing remnant nephrectomy, diabetic nephropathy, and sepsis-induced acute kidney injury had a similar profibrotic role of ALOX15 [52–54]. Consistent with these studies, the ALOX15 expression levels were found to be significantly higher in the kidneys of patients with advanced diabetic nephropathy, one of the main complications of diabetes [55]. This particular study focused on the glomeruli in patients, where ALOX15 levels were elevated in all intrinsic cells of glomerulus and contributed to ferroptosis induction mainly through the ALOX15-mediated lipid metabolism pathway. Compared to the consistent in vivo results in mice, the role of ALOX15 in vitro studies in mouse macrophages is a bit more complicated. Indeed, we have not been able to detect both mRNA and protein expressions of ALOX15 in BMDMs. This result is consistent with previous reports [56, 57] and may be one reason why ALOX15 was not determined as a common M2 macrophage marker for both mice and humans in the Martinez’s report [14]. Since ALOX15 expression in macrophage is detected in vivo (Fig. 8), a possible problem is that in vitro experiments using BMDMs do not adequately reflect the in vivo situation of renal fibrosis. The resident (peritoneal) macrophages have been reported to have a larger population of ALOX15-positive cells compared to BMDMs [58], suggesting that TG2 in BMDMs may involves in not only its own M2 polarization but also that of resident macrophages during renal fibrosis.

In this study, we found that TG2 promoted M2 macrophage polarization via induction of ALOX15 expression, although the regulatory mechanism of TG2 and ALOX15 expression remains to be clarified. Phenformin, a biguanide antidiabetic drug, was reported to prevent IL-4-induced M2 macrophage polarization through decreased STAT6 association and Lys-9 acetylation of histone H3 at the ALOX15 promoter [59]. Since STAT6 is also implicated to associate at the TG2 promoter [60], we demonstrated that an inhibitor of STAT6 phosphorylation predominantly suppressed TG2 expression (Suppl. Fig. S6), whereas TG2 inhibitors did not interfere the phosphorylation and expression of STAT6 (Fig. 7A). Mitogen-activated protein kinase kinase (MEK) is required for M2 macrophage polarization by promoting PPARγ-induced retinoic acid signaling [49]. PPARγ and retinoic acid signaling have also been reported as upstream regulators of TG2 [61] although PPARγ is also reported to be inactivated via crosslinking by TG2 [62, 63]. Moreover, in IL-4-induced M2 macrophages, ALOX15 generates an endogenous ligand for the transcription factor PPARγ, thereby suppressing inflammatory responses [40]. Knockdown and inactivation of TG2 reduced the expression of ALOX15 and vice versa, suggesting that TG2 and ALOX15 expressions were synergistically upregulated for M2 macrophage polarization. In addition to MEK, histone deacetylase activity is also critical for M2 macrophage polarization [49]. Demethylation of histone H3 trimethyl-lysine 27 (H3K27me3) at the promoter is required for IL-4-mediated ALOX15 induction [64]. Furthermore, TG2 serotonylates the glutamine at position 5 (Q5ser) on nucleosomes marked with tri-methylated lysine 4 of histone H3 (H3K4me3) and is responsible for enhanced recruitment of transcription factor, TFIID [65]. Since we detected the increased level of nuclear TG2 in IL-4-treated human macrophage (Suppl. Fig. 7), TG2-mediated serotonylation and regulations of enzyme involved in methylation/demethylation of histone H3 may contribute to the various gene expressions. In addition, we attempted to detect the direct molecular target of TG2 in IL-4-treated human macrophage based on the incorporation of a biotin-pentylamine (BPA) probe into proteins via crosslinking activity. However, the crosslinking activity is weaker than the background signal (due to nonspecific adsorption of streptavidin-peroxidase and the several endogenous biotin-binding proteins) and cannot be detected (data not shown). Based on these results, we speculated that it may be difficult to detect TG2 activity using BPA in cultured macrophages because BPA does not penetrate human macrophage cell membranes, is quickly excreted, or the BPA-incorporated proteins are rapidly degraded. However, the experiments using cell lysates of IL-4-treated human macrophages showed an increase in BPA-incorporated proteins (Suppl. Fig. 8). The detailed relationship between TG2 and ALOX15 needs to be clarified in future studies.

In summary, we demonstrated that a TG2-dependent M2 macrophage polarization mechanism was commonly induced in both mouse and human cells and was involved in renal fibrosis in cellular and animal models. Furthermore, we found that ALOX15 was an important factor acting in the downstream of intracellular TG2 activity in the polarization of M2 macrophages and exacerbated renal fibrosis (Fig. 8C). Our findings provide information about novel pathological mechanisms and new therapeutic targets associated with renal fibrosis that could be widely adopted to several research fields related to TG2 and macrophage polarization.

## Supplementary information

Supplemental materials and original data files

Supplementary Tables

## Data Availability

All data generated or analyzed in this study are available from the corresponding author upon reasonable request.
