# Supplementary material for: Tissue transglutaminase exacerbates renal fibrosis via alternative activation of monocyte-derived macrophages
Source: Cell Death Dis. 2023 Mar 2;14(2):136. doi: 10.1038/s41419-023-05622-5 (PMC9981766; doi:10.1038/s41419-023-05622-5)
Supplement: Supplementary file 1 — Supplemental materials and original data files [file 41419_2023_5622_MOESM1_ESM.pdf]

## Supplementary Material

Suppl Fig. S1

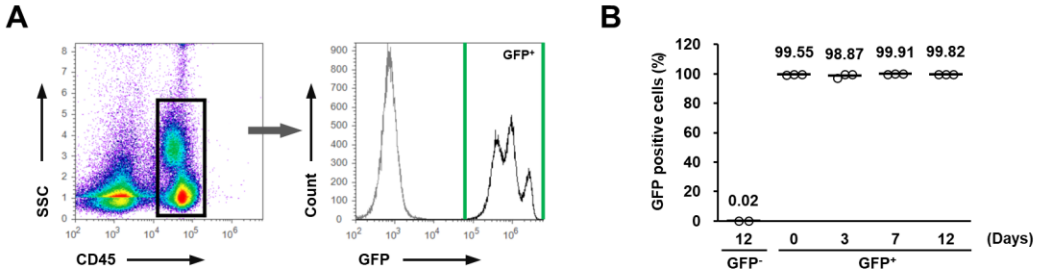


**Suppl Figure S1. Evaluation of replacement rates in bone marrow transplantation experiments.**

Mice were irradiated at lethal dose (8.5 Gy) of X-rays and transplanted with bone marrow cells isolated from GFP-transgenic mice by tail vein injection. After 4 weeks recovery period, mice were subjected to UUO surgery and analyzed on indicated days. Peripheral blood cells in UUO-treated mice were collected, divided into SSC and CD45, populations indicated by square line was classified by GFP expression (A). The gray line in histogram plot in A was indicated as negative control that mice were transplanted with bone marrow cells from normal WT mice (GFP$^-$). The percentage of GFP$^+$ cells in CD45$^+$ blood cells on indicated days after UUO surgery were presented (B).

**Suppl Fig. S2**

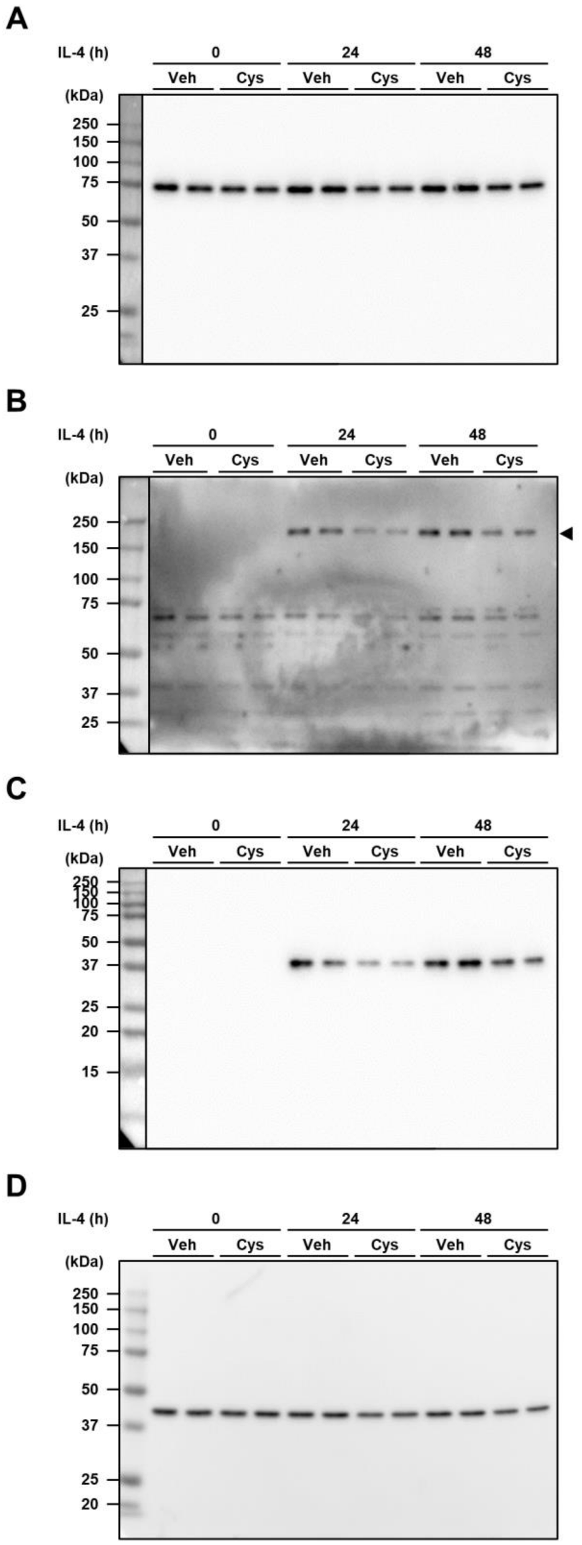


**Suppl Figure S2. Full-length and uncropped blots from Fig. 4C.**

The data of western blotting analyses in Fig 4C were cropped for presentation. The blots with the lysates from bone marrow-derived macrophages were analyzed by immunoblotting using anti-TG2 (A), CD206 (B), Arg-1 (C) antibodies. The blot using anti-β-actin antibody was used as loading controls in each sample (D).

**Suppl Fig. S3**

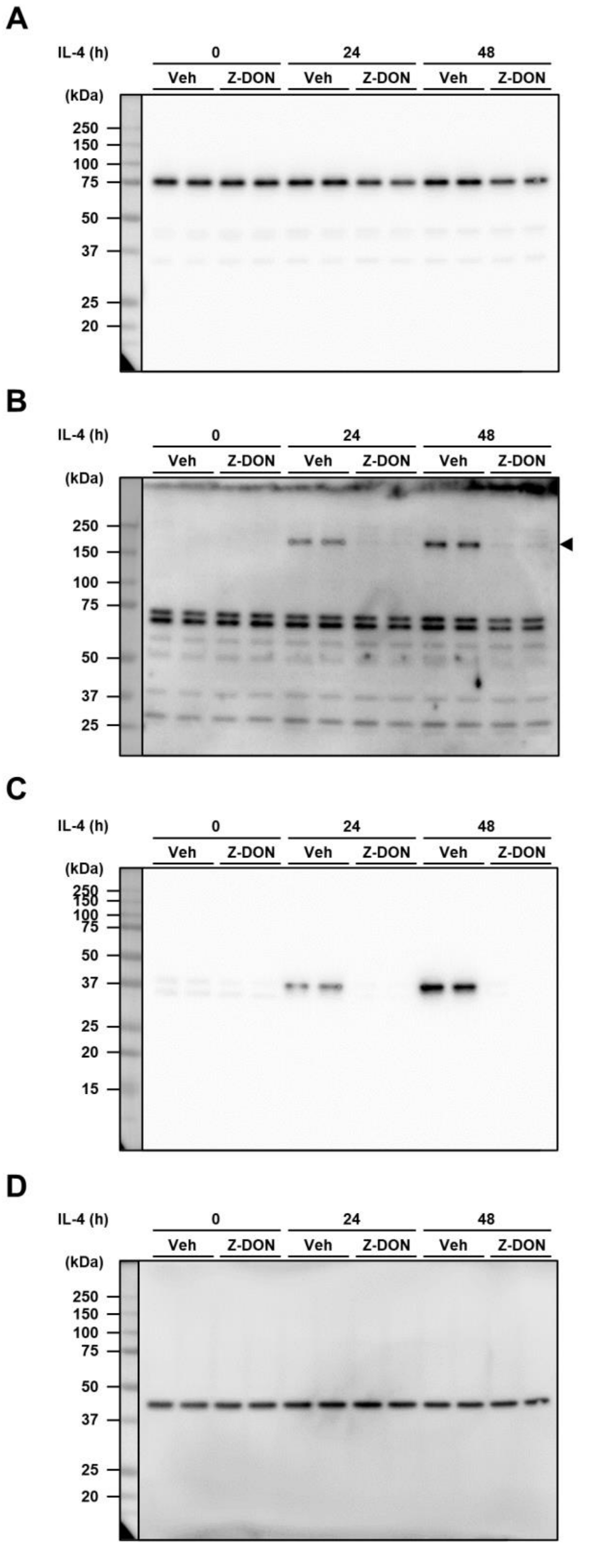


**Suppl Figure S3. Full-length and uncropped blots from Fig. 4D.**

The data of western blotting analyses in Fig 4D were cropped for presentation. The blots with the lysates from bone marrow-derived macrophages were analyzed by immunoblotting using anti-TG2 (A), CD206 (B), Arg-1 (C) antibodies. The blot using anti-β-actin antibody was used as loading controls in each sample (D).

**Suppl Fig. S4**

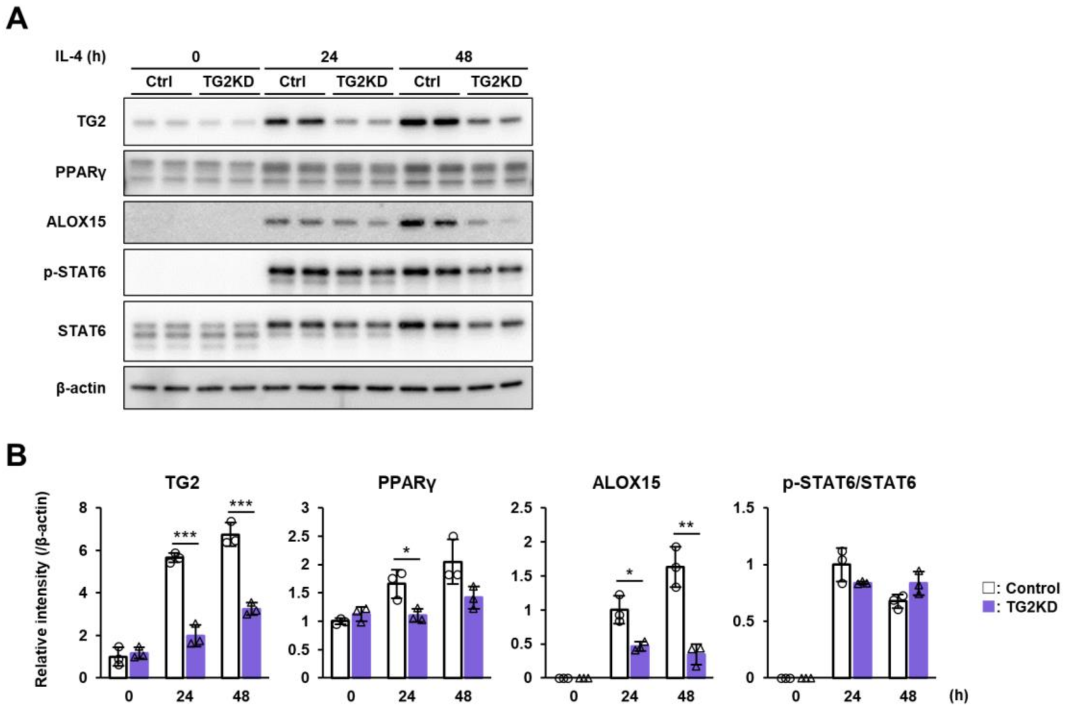


**Suppl Figure S4. Evaluation of various related protein in IL-4-treated THP-1-derived macrophages.**

Human monocytic leukemia cell line, THP-1, was treated with 150 nM PMA for 24 h. PMA-treated macrophages were transfected with siRNA against TG2 and treated with 20 ng/ml recombinant human IL-4. Protein levels of TG2, PPARγ, ALOX15, p-STAT6, and STAT6 were analyzed (A). As a negative control, scrambled siRNA (Control) was replaced with the same amount of TG2 siRNA. Total intensities of all the bands in each sample were presented after normalizing the results to the expression levels in β-actin (B). The intensities of p-STAT6 were normalized to the expression levels in STAT-6. Relative values were presented as the mean ± SD (n = 3) (***$P < 0.001$, **$P < 0.01$, *$P < 0.05$, Student's t-test).

**Suppl Fig. S5**

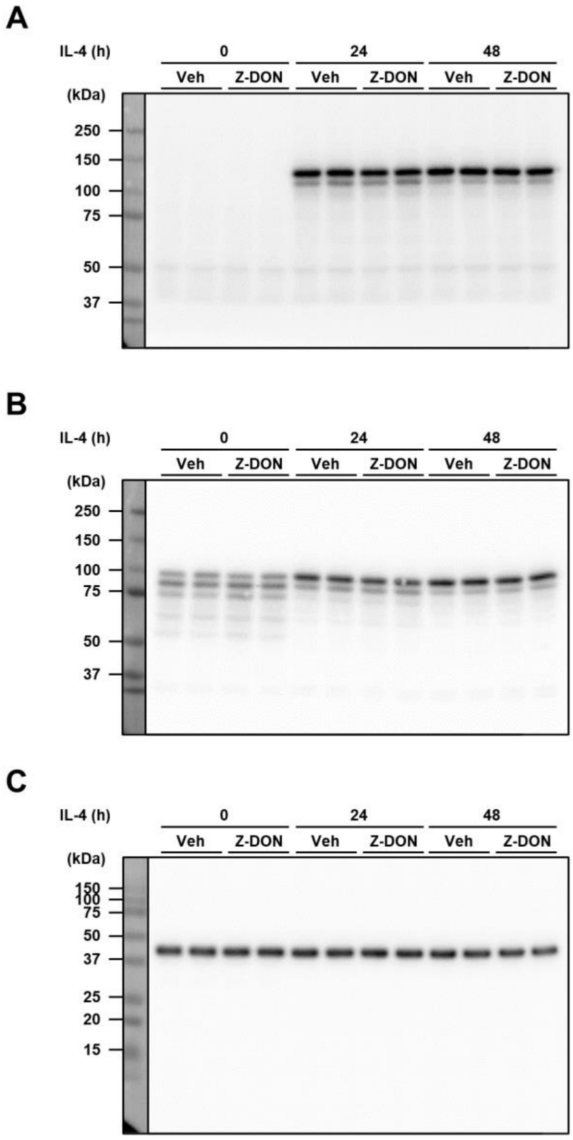


**Suppl Figure S5. Full-length and uncropped blots from Fig. 7A.**

The data of western blotting analyses in Fig 7A were cropped for presentation. The blots with the lysates from THP-1-derived macrophages were analyzed by immunoblotting using anti-phosphorylated STAT6 (A) and STAT-6 (B) antibodies. The blot using anti-β-actin antibody was used as loading controls in each sample (C).

## Suppl Fig. S6

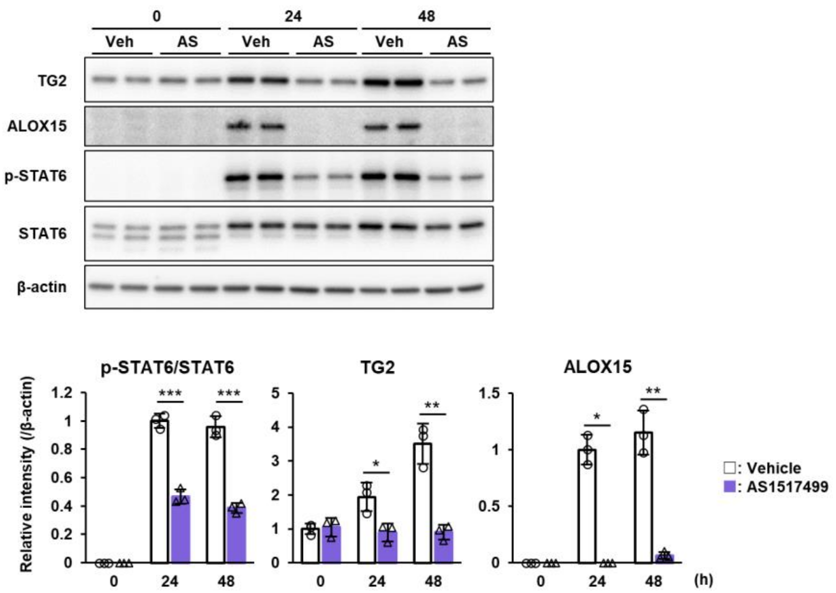


**Suppl Figure S6. STAT6 inhibitor decreased TG2 expression in THP-1-derived M2 macrophage.**

THP-1 was treated with 150 nM PMA for 24 h. M2 macrophage polarization was induced by 20 ng/ml recombinant human IL-4 in the presence or absence of STAT6 inhibitor (250 nM AS1517499). The protein levels of these samples were analyzed by immunoblotting using anti-TG2, phosphorylated STAT6, and STAT-6 antibodies. The blot using anti-β-actin antibody was used as loading controls in each sample. (****P* < 0.001, ***P* < 0.01, **P* < 0.05, Student's t-test).

Suppl Fig. S7

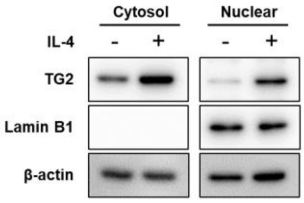


**Suppl Figure S7. Subcellular localization of TG2 in THP-1-derived M2 macrophage.**

THP-1 was treated with 150 nM PMA for 24 h. M2 macrophage polarization was induced by 20 ng/ml recombinant human IL-4. The cytosol and nuclear fractions were separated using Minute Cytoplasmic and Nuclear Fractionation kit (Invent Biotechnologies). The protein levels of these samples were analyzed by immunoblotting using anti-TG2 antibody. Anti-Lamin B1 antibody was used as a loading control for nuclear fractions. Anti-β-actin antibody was used as loading controls in each sample.

Suppl Fig. S8

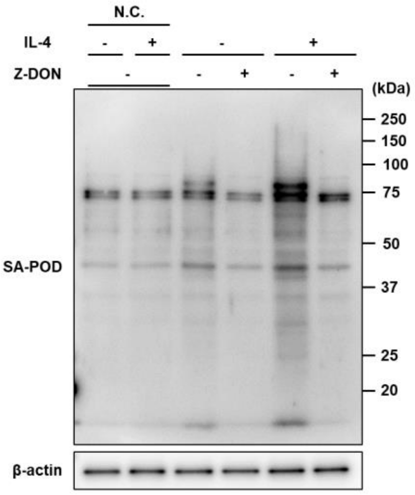


**Suppl Figure S8. Detection of TG2 substrate proteins crosslinked with biotin-pentylamine in THP-1-derived M2 macrophage.**

THP-1-derived M2 macrophage was treated with 150 nM PMA for 24 h and 20 ng/ml human IL-4. The cell lysates were homogenized in lysis buffer containing 50 mM Tris-HCl (pH 8.0), 150 mM NaCl, 1 mM EDTA, 1% NP-40, and protease inhibitor cocktail. Each soluble extract (25 μg) was incubated with (100 μM) biotin-pentylamine (BPA) with 5 mM $CaCl_2$ and 1 mM DTT in the presence of absence of 50 μM Z-DON, and the amounts of Gln-donor substrates incorporated with BPA were detected by Western blot using peroxidase-conjugated streptavidin (SA-POD). Anti-β-actin antibody was used as a loading control for each sample. Negative Control (N.C.) included the addition of 25 mM EDTA during the incubation for crosslinking reactions.
