# Supplementary material for: Tissue transglutaminase exacerbates renal fibrosis via alternative activation of monocyte-derived macrophages
Source: Cell Death Dis. 2023 Mar 2;14(2):136. doi: 10.1038/s41419-023-05622-5 (PMC9981766; doi:10.1038/s41419-023-05622-5)
Supplement: Supplementary file 2 — Supplementary Tables [file 41419_2023_5622_MOESM2_ESM.docx]

**Supplementary Tables**

**Suppl. Table S1. Antibodies used for western blotting, immunostaining, and flow cytometry analyses**

| **Antibodies** | **Source** | **Identifier** | **Dilution** |
| --- | --- | --- | --- |
| ***For western blotting and immunostaining*** |  |  |  |
| Rabbit anti-mouse TG2 antibody | Produced in house |  | 1/4000 |
| Rat anti-mouse F4/80 (CI:A3-1) | Bio-rad | MCA497G | 1/500 |
| Rabbit anti-α-SMA antibody | Abcam | Ab5694 | 1/200 |
| Rabbit anti-CD206 antibody | Proteintech | 18704-1-AP | 1/500 |
| Anti-arginase 1/ARG1 antibody (Clone: E-2) | Santa Cruz | sc-271430 | 1/500 |
| Anti-β-Actin antibody (Clone: 8H10D10) | Cell Signaling technology | #3700 | 1/10000 |
| Anti-Stat6 antibody | Cell Signaling technology | #9362 | 1/1000 |
| Anti-p-Stat6 Antibody (pY641.18) | Santa Cruz | sc-136019 | 1/500 |
| Anti-15 lipoxygenase 1 antibody (Clone: EPR22138) | Abcam | Ab244205 | 1/500 |
| AlexaFluor 488 goat anti-rabbit IgG | Invitrogen | A-11008 | 1/2000 |
| AlexaFluor 594 chicken anti-rat IgG | Invitrogen | A-21471 | 1/2000 |
|  |  |  |  |
| ***For flow cytometry*** |  |  |  |
| APC anti-CD206 (MMR) antibody (Clone: MR6F3) | Invitrogen | 17-2061-80 |  |
| APC/Cy7 anti-mouse/human CD11b Antibody | Biolegend | 101226 |  |
| FITC anti-mouse F4/80 Antibody | Biolegend | 123107 |  |
| PerCP-Cyanine5.5 anti-CD45 antibody (Clone: 30-F11) | Invitrogen | 45-0451-80 |  |
| PE/Cyanine7 anti-mouse F4/80 Antibody | Biolegend | 123113 |  |
| PE-Cyanine7 anti-Ly-6C antibody (Clone: HK1.4) | Invitrogen | 25-5932-80 |  |

**Suppl. Table S2. Primer pairs for RT-qPCR experiments**

| **Species** | **Gene** | **Forward** | **Reverse** | **Product size** |
| --- | --- | --- | --- | --- |
| Mouse | *Arg-1* | CTCCAAGCCAAAGTCCTTAGAG | AGGAGCTGTCATTAGGGACATC | 185 |
|  | *CD36* | ATGGGCTGTGATCGGAACTG | GTCTTCTCAATAAGCATGTCTCC | 110 |
|  | *CD206* | CGGTGAACCAAATAATTACCAAAAT | GTGGAGCAGGTGTGGGCT | 158 |
|  | *TBP* | ACCGTGAATCTTGGCTGTAAAC | CGCAGTTGTCCGTGGCTCTC | 123 |
|  | *TFR* | CCAGTGTGGGAACAGGTCTT | ATAAGGGGCTGGCAGAAACT | 249 |
|  | *TG2* | AGCCGATGATGTGTACCTAG | AGGATTCCATCCTCGAACTG | 138 |
|  |  |  |  |  |
| Human | *TG2* | ATGCCGACGTGGTAGACTGG | CACTGCCCATGTTCATGCTC | 270 |
|  | *PPARγ* | TGACCAGAAGCCTGCATTTCTG | TCCACGGAGCTGATCCCAAAG | 186 |
|  | *ALOX15* | CAACCACCAAGGATGCAACG | CCCACAGCCACCATAACGG | 124 |
|  | *CCL22* | TACTGGACCTCAGACTCCTGC | CCACGGTCATCAGAGTAGGC | 146 |
|  | *CD209* | GGGAGAGTGGGGTGACATGAG | CCAAGAGCGTGAAGGAGAGG | 176 |
|  | *CD36* | CAAAACGGCTGCAGGTCAAC | TTCTCATCACCAATGGTCCCAG | 127 |
|  | *TBP* | TCAAACCCAGAATTGTTCTCCTTAT | CCTGAATCCCTTTAGAATAGGGTAGA | 122 |
|  | *MCP-1* | GATCTCAGTGCAGAGGCTCG | TTTGCTTGTCCAGGTGGTCC | 155 |
|  | *CXCL10* | TGCCATTCTGATTTGCTGCC | CAGTTCTAGAGAGAGGTACTCCTTG | 71 |
